# Oxytocin in pig seminal plasma is positively related with *in vivo* fertility of inseminated sows

**DOI:** 10.1186/s40104-021-00620-z

**Published:** 2021-09-13

**Authors:** Lorena Padilla, Marina López-Arjona, Silvia Martinez-Subiela, Heriberto Rodriguez-Martinez, Jordi Roca, Isabel Barranco

**Affiliations:** 1 Department of Medicine and Animal Surgery, Faculty of Veterinary Medicine, University of Murcia, E-30100 Murcia, Spain; 2 Department of Biomedical & Clinical Sciences (BKV), BKH/Obstetrics & Gynaecology, Faculty of Medicine and Health Sciences, Linköping University, SE-58185 Linköping, Sweden; 3 Department of Veterinary Medical Sciences, University of Bologna, IT-40064 Ozzano dell’Emilia, Bologna, Italy

**Keywords:** Artificial insemination, Ejaculate, Fertility, Oxytocin, Pig, Semen quality, Seminal plasma

## Abstract

**Background:**

Identification of relevant *in vivo* biomarkers for fertility remains a challenge for the livestock industry. Concentrations of the small peptide hormone oxytocin (OXT), involved in male reproductive function and present in the seminal plasma (SP) of several species could be a robust one. This study characterized concentrations of SP-OXT in ejaculates from boars used in artificial insemination (AI) programs aiming to evaluate its relationship with sperm quality variables and *in vivo* fertility of their liquid-stored AI-semen. Seminal OXT concentrations (ng/mL) were measured in 169 ejaculates from 61 boars of the Duroc, Pietrain, Landrace and Large White breeds using a direct competitive immunoassay test based on AlphaLISA^®^ technology. Ejaculate (ejaculate volume, sperm concentration, total sperm count) and sperm parameters (motility, viability, intracellular generation of reactive oxygen species, plasma membrane fluidity) were assessed at 0 h and 72 h in AI-semen samples stored at 17 °C. *In vivo* fertility included only 18 Large White and Landrace boars whose AI-semen was used to inseminated > 100 sows and evaluated both farrowing rate and litter size of 3,167 sows.

**Results:**

The results showed that SP-OXT differed between boars and between ejaculates within boar (*P* < 0.05) but not between breeds (Duroc, Pietrain, Landrace and Large White). Ejaculates with higher SP-OXT concentration/mL (hierarchically grouped; *P* < 0.001) had larger volume and came from younger boars (*P* < 0.05). Ejaculates of boars showing positive farrowing rate deviation exhibited higher (*P* < 0.05) SP-OXT concentration/mL than those with negative farrowing rate deviation.

**Conclusion:**

The SP concentrations of OXT are boar, ejaculate and age dependent, and positively related with ejaculate volume and farrowing rates of liquid-stored semen AI-doses.

**Supplementary Information:**

The online version contains supplementary material available at 10.1186/s40104-021-00620-z.

## Background

Artificial insemination (AI) is the most widespread and effective reproductive technology for the production and genetic improvement of pigs worldwide [1–3]. To improve AI-efficiency, the latest trends point towards to a reduction in both the sperm numbers per AI-dose and the number of AIs performed per sow, which will lead to more than 6,000 sows being inseminated each year with semen from a single boar [4, 5]. However, there are still drawbacks in meeting this challenge, one of the most important ones being the wide variability among boars in the ability of sperm to withstand preservation, either at liquid or frozen state, as well as on *in vivo* fertility rates after AI [4, 6, 7]. This situation evidences that the conventional semen analyses routinely used in AI centers for selecting boars and/or ejaculates are not reliable enough to foresee the reproductive performance of seminal AI-doses [4]. Therefore, one of the current challenges of the swine industry focuses on discovering biomarkers that would allow a more precise selection of fertile boars.

The last studies in this realm have proposed searching for these biomarkers in seminal plasma (SP), a protein rich-fluid composed mainly by secretions from epididymis and accessory sex glands [8]. Certainly, the complex composition of SP together with its essential roles for sperm function as well as in the successful reproductive performance of female genital tract, make SP a valuable source for eventual male fertility biomarkers [9–12]. Although the specific components of SP involved in these reproductive events have not yet been fully identified, proteins and peptides would be among the major contributors [8]. Indeed, recent studies have highlighted some SP proteins and peptides as putative biomarkers for sperm functional capacity and *in vivo* fertility outcomes of pig semen AI-doses [13–16]. For instance, glutathione peroxidase-5 [13, 17], anti-Müllerian hormone [18] or transforming growth factor-1 [19].

Oxytocin (OXT), a small peptide hormone mainly synthetized in the magnocellular neurons of the hypothalamus, would be another SP component potentially involved in male fertility. Local OXT synthesis and the presence of OXT receptor (OXTR) have been located in the male reproductive tract of several mammalian species (pig: [20]; cow: [21]; rat: [22]; horse: [23]; sheep: [24]; human: [25]). Moreover, the presence of OXT in semen has also been documented in men [26, 27] and stallions [23] and a putative role of SP-OXT on both sperm functionality and female genital tract performance has been suggested [27]. As far as we are aware of, no studies have been conducted in any livestock species evaluating the putative relationship between SP-OXT with sperm quality and function and *in vivo *fertility after AI.

In pigs, the main role attributed to OXT is to facilitate the contractility of the myometrium [28]. Consequently, OXT is sometimes used as an additive to semen AI-doses in order to accelerate the progression of semen towards the uterus for better colonization of the uterine-tubal reservoir [29]. Improvements in fertility, both in terms of farrowing rates and litter size, have been documented in sows inseminated with semen doses supplemented with OXT [30–34]. These results open the hypothesis that seminal OXT could influence the fertility outcomes of pig AI-semen. Accordingly, the present study aimed to, apparently for the first time, (1) characterize the concentrations of OXT in pig-SP and (2) evaluate the putative relationship between SP-OXT concentrations with sperm quality and function as well as with *in vivo* fertility AI-outcomes.

## Methods

### Reagents

Unless otherwise specified, reagents and equipment for OXT measurements were from PerkinElmer (Waltham, MA, USA) and fluorochromes for sperm analyses from Thermo Fisher Scientific (Waltham, MA, USA). The extender used for the AI-semen doses was Biosem+ (Magapor, Zaragoza, Spain), declared free from exogenous/added hormones. Phosphate buffered saline (PBS; Merck KgaA, Darmstadt, Germany) was used to extend semen samples for flow cytometry analyses.

### Boars, ejaculates and seminal plasma

Entire ejaculates were collected from mature (ranging from 9 to 35 months [mos.]) and healthy boars of different breeds (Landrace, Large White, Duroc and Pietrain) included in AI-programs. The boars were housed in a Spanish AI-center (AIM Ibérica; Topigs Norsvin Spain SLU, Calasparra, Murcia, Spain), that complied with the Spanish and European rules for both animal health and welfare and for the marketing of seminal AI-doses (Spanish: ES300130640127, August 2006; European: ES13RS04P, July 2012). The boars were placed in individual pens in a building with controlled air temperature (15–25 °C) and with 16 h of continual light per day. Boars were free of chromosomal translocations and with percentages of spermatozoa with fragmented nuclear DNA lower than 3% (measured using the sperm chromatin stability assay, following the procedure described by Martinez-Pastor et al., [35]). Boars were subjected to a regular ejaculate collection (twice weekly) to produce semen AI-doses. Entire ejaculates were collected using a semi-automatic collection method (Collectis^®^, IMV Technologies, L’Aigle, France) and those used in the experiments exceeded the quantity and sperm quality thresholds for production of semen AI-doses. Specifically, more than 200 × 10^6^ sperm/mL, 70% motile sperm and 75% sperm with normal morphology.

The SP was harvested immediately after ejaculate collection. From each ejaculate, a semen sample of 5 mL was centrifugated twice at room temperature at change to: 1,500 × *g* for 10 min (RT, Rotofix 32A, Hettich Centrifuge UK, Newport Pagnell, Buckinghamshire, England, UK). The harvested SP was microscopically examined (Eclipse E400; Nikon, Tokyo, Japan) to check for sperm presence. The SP samples were stored in 3 mL-cryotubes at − 80 °C (Ultra Low Freezer; Haier Inc., Qingdao, China) until OXT measurement.

### Measurement of SP-OXT

**Fig. 1:**
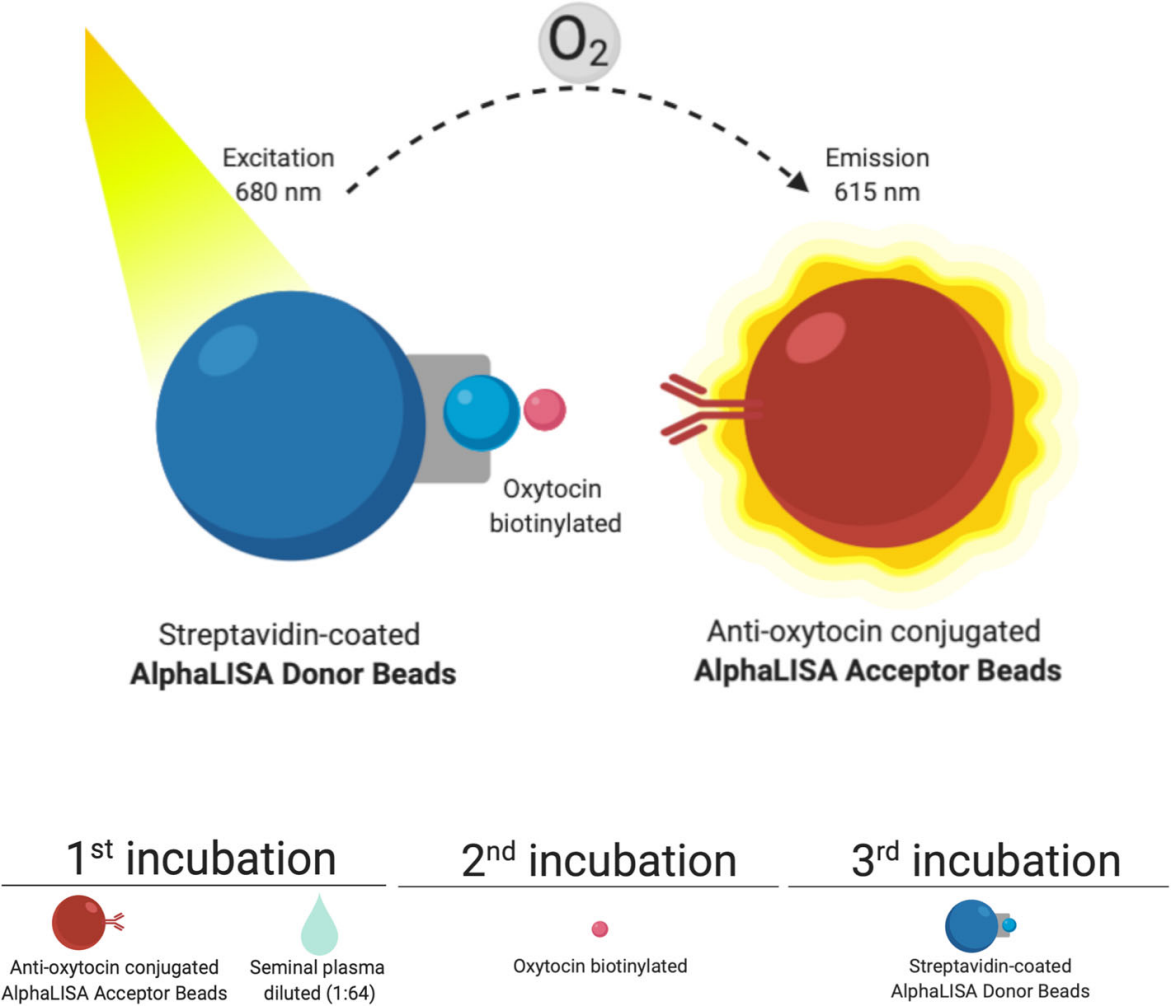
Direct competitive immunoassay test based on AlphaLISA^®^ used for oxytocin assessment in pig seminal plasma samples

Seminal OXT was measured using a direct competitive immunoassay test based on AlphaLISA^®^ technology, adapted to pig SP samples, using a monoclonal anti-oxytocin antibody highly specific for OXT [36] (Fig. 1). Firstly, the frozen SP was thawed on ice and diluted in AlphaLISA^®^ Universal buffer (1:64; v/v). A standard eight-points curve (ranging from 0 to 4,800 pg/mL) was generated using OXT conjugated to bovine serum albumin. For each standard and diluted SP-samples, a 15 μL aliquot was added, in duplicate, into the appropriate wells. Then, 15 μL of acceptor beads coated with monoclonal anti-oxytocin antibody (30 μg/mL) was added to each well and the plates were incubated at RT in darkness for 90 min. Thereafter, 10 μL of biotinylated OXT-BSA (1 nmol/L, Cusabio Technology LLC, Houston, TX, USA) was added to each well and the plates incubated again at RT for 60 min. Finally, 10 μL of donor beads (20 μg/mL) was added to each well and the plates incubated at RT in darkness for 30 min. The fluorescence intensity was measured through the EnSpire Multimode Plate Reader. The intraassay variation was below 7.5% and the inter-assay coefficient variation below 9%, the assays displaying also high linearity under serial dilutions. The SP-OXT concentrations were expressed in ng/mL.

### Assessment of sperm quanti- and qualitative parameters

The parameters assessed were: (1) ejaculate volume, (2) sperm concentration, (3) total sperm count, (4) sperm morphology, (5) sperm motility (total and progressive), (6) integrity of plasma and acrosomal sperm membranes (sperm viability), (7) intracellular generation of reactive oxygen species (ROS) by viable spermatozoa and (8) membrane lipid disorder of viable spermatozoa. Ejaculate volume (mL) was measured by depositing the freshly collected ejaculate in a graduated cylinder while sperm concentration (× 10^6^ sperm/mL) was measured with an automated cell counter (NucleoCounter^®^ NC-100TM; ChemoMetec, Allerod, Denmark). Total sperm count was calculated by multiplying ejaculate volume by sperm concentration. Sperm morphology was microscopically (Eclipse E400, Nikon, Tokyo, Japan) assessed in fixed sperm samples (1:1, v/v, in 0.12% formaldehyde saline solution; Panreac, Barcelona, Spain) by counting a total of 200 sperm per sample.

Sperm functional parameters were assessed in semen samples extended in the same Biosem+ extender used to prepare AI-doses, to a final concentration of 30 × 10^6^ sperm/mL. Sperm motility was evaluated using a computer assisted sperm analyzer (CASA, ISASV1^®^, Proiser R + D S.L., Paterna, Spain). Briefly, 5 μL-aliquots of diluted semen samples were placed in a Makler chamber (Sefi Medical Instruments, Haifa, Israel) pre-warmed at 38 °C. Six to ten fields were analyzed, accumulating data of more than 600 spermatozoa per semen sample. The percentage of spermatozoa with an average path velocity greater than 20 μm/s were considered as motile and those with a straight-line velocity more than 40 μm/s were recorded as showing progressive motility. The other sperm parameters were assessed by flow cytometry (BD FACS Canto II flow cytometer, Becton Dickinson & Company, Franklin Lakes, NJ, USA). Three technical replicates (with 10,000 events positive to Hoechst 33342 [H-42]) were analyzed for each semen sample. For sperm viability, semen aliquots of 100 μL were incubated with 3 μL H-42 (0.05 mg/mL in PBS), 2 μL propidium iodide (PI, 0.5 mg/mL in PBS) and 2 μL fluorescein-conjugated peanut agglutinin (PNA-FITC, 100 μg/mL in PBS) during 10 min at 37 °C in darkness (Sanyo MIR-153 incubator, Gemini BV, Apeldoorn, Netherlands). Immediately before to flow cytometry analysis, 400 μL of PBS were added to each sample. The percentage of spermatozoa with intact plasma and acrosome membranes (H-42+/PI−/PNA-FITC-) were recorded as viable sperm. Intracellular ROS generation in viable sperm was measured in semen samples of 50 μL extended in 950 μL of PBS and incubated with 1.5 μL of H-42 (0.05 mg/mL in PBS), 1 μL of PI (0.5 mg/mL in PBS), and 1 μL of 5- (and 6-) chloromethyl-20,70-dichlorodihydrofluorescein diacetate acetyl ester (CM-H_2_DCFDA, 1 mmol/L in dimethyl sulfoxide [DMSO]) at 38 °C in darkness for 30 min. The percentage of viable sperm exhibiting high intracellular ROS generation (H-42+/PI−/2′,7′-di-chlorofluorescein [DCF]+) was recorded. Sperm membrane lipid disorders were assessed in 50 μL semen samples extended in 950 μL of PBS and incubated with 2.5 μL of H-42 (0.05 mg/mL in PBS) and 10 μL of Yo-Pro-1 (2.5 μmol/L in DMSO) at 38 °C in darkness for 8 min. After this incubation time, 26 μL of Merocyanine 540 (M-540, 0.1 mmol/L in DMSO) was added to each semen sample and incubated for at 38 °C in darkness another 2 min. The percentage of viable spermatozoa exhibiting high plasma membrane fluidity (H-42+/ Yo-Pro-1−/M-540+) was recorded.

### Experimental design

#### Experiment 1: characterization of OXT concentration/mL in boar SP. inter-breed, inter-boar and intra-boar variability in SP-OXT concentrations

To investigate variability between breeds, SP was harvested from the ejaculates (one per boar) of 61 boars from Duroc (*n* = 14), Pietrain (*n* = 18), Landrace (*n* = 15) and Large White (*n* = 14) breeds. To evaluate the variability among boars (inter-boar variability) and among the ejaculates from the same boar (intra-boar variability), SP samples from ejaculates (four samples collected from four ejaculates per boar) of 18 boars from Large White (*n* = 5) and Landrace (*n* = 13) breeds were analyzed.

#### Experiment 2: relationship between SP-OXT concentration and boar ejaculate characteristics

A total of 36 ejaculates (one per boar) collected from boars of different ages (from 9 to 35 months) of the same mentioned above breeds were used. Once the ejaculate volume was measured, three 5 mL semen aliquots were collected from each ejaculate to: (1) measure sperm concentration and evaluate sperm morphology; (2) evaluate sperm functional parameters once extended to 30 × 10^6^ sperm/mL and stored at 17 °C for 72 h (analyzed at 0 h and 72 h of storage time); and (3) harvest SP samples for OXT measurement.

#### Experiment 3: relationship between SP-OXT concentration in the original ejaculate and *in vivo* fertility outcomes of extended semen samples

A total of 72 SP samples were collected for OXT measurement from ejaculates of 18 Landrace (*n* = 13) and Large White (*n* = 5) boars (four ejaculates per boar) over 12-month period (one SP sample per boar every four mos. to avoid possible seasonal effects). During this 12-month period, the ejaculates of these boars were used to produce liquid-storage semen AI-doses (2,400 × 10^6^ of total spermatozoa in 80 mL). These semen AI-doses were used to cervically inseminate twice per estrus a total of 3,167 multiparous (1–5 farrowing) Landrace and Large White sows (> 100 sows inseminated per boar). The sows were housed in different Spanish farms but subjected to the same management system. The fertility outcomes of inseminated sows were recorded in terms of (1) percentage of farrowing sows over total inseminated (farrowing rates) and (2) the total number of piglets born per litter (litter size).

### Statistical analysis

Statistical analyses were performed with GraphPad Prism 9.0 (GraphPad Software, Inc., CA, US) and IBM SPSS Statistics 24.0 (IBM, Armonk, NY, USA). Firstly, Shapiro–Wilk test was used to evaluate the assumption of normality in the residual data for each statistical parameter. In Experiment 1, one-way ANOVA was used to investigate inter-breed, inter-boar variability on SP-OXT concentrations and intra-boar reliability was assessed by intra-class correlation (ICC) in a two-way mixed approach (ICC 3,1). In Experiment 2, Spearman correlation coefficients between SP-OXT and ejaculate characteristics were calculated. A hierarchical cluster analysis was performed to classify ejaculates into groups according to seminal OXT concentration and two groups were generated, one with higher and other with lower SP-OXT samples. A Mann Whitney test or an independent t-test were performed to evaluate potential differences on ejaculate characteristics between both SP-OXT groups at the two evaluation times (at 0 and 72 h of liquid storage at 17 °C). In Experiment 3, a multivariate statistical model [37] was applied to the *in vivo* fertility records in order to adjust them for parameters related to farm and sow and to identify the direct boar effect for farrowing rate and litter size. A hierarchical cluster analysis was performed to classify the boars into groups according to fertility outcomes. Three groups were generated for both farrowing rate and litter size. The groups grouped the boars as having positive deviation, without deviation and negative deviation with respect to the mean fertility outcomes of the totality of boars of the same breed. One-way ANOVA was carried to evaluate potential differences on SP-OXT concentration between the three groups. Statistical differences were defined as *P* < 0.05. Results from non-normality distribution data were shown as median (25^th^ and 75^th^ percentiles), and those from normality distribution data as mean ± standard error of the mean (SEM).

## Results

### Experiment 1: characterization of SP-OXT concentration. Inter-breed, inter-boar and intra-boar variability in SP-OXT concentrations

**Fig. 2:**
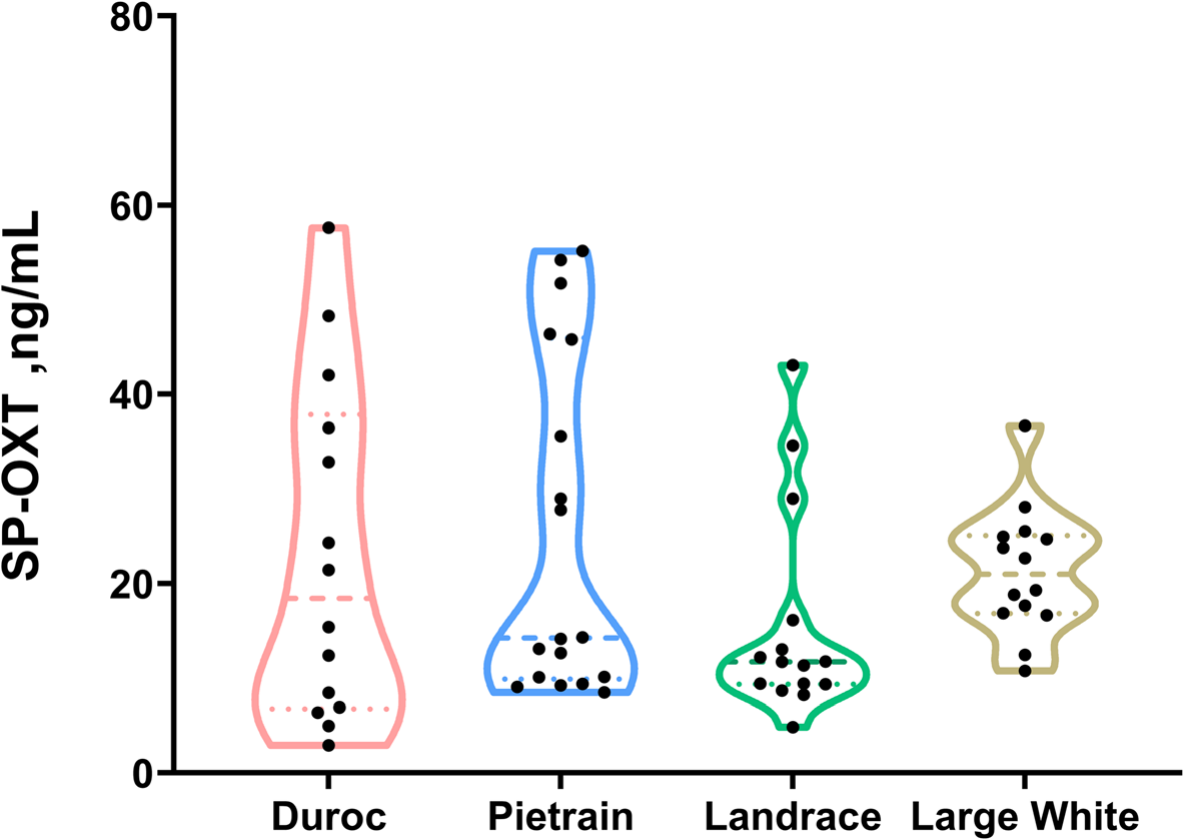
Violin plots showing the oxytocin (OXT) concentration measured in seminal plasma (SP) samples from entire ejaculates (*n* = 61) collected from boars of different breeds (Duroc, *n* = 14; Pietrain, *n* = 18; Landrace, *n* = 15; Large White, *n* = 14) used as semen donors in artificial insemination programs. Each dot indicates SP-OXT concentration of each individual ejaculate, dashed lines the median and dotted lines the 25^th^ and 75^th^ percentiles. No differences (*P* > 0.05) were observed among breeds in SP-OXT concentration

The SP concentration of OXT did not differ among breeds (inter-breed variability), the four breeds showing similar concentrations (median, 25−75^th^ percentiles; Pietrain: 14.23, 9.91–45.95 ng/mL; Duroc: 18.43, 6.78–37.86 ng/mL; Landrace: 11.70, 9.36–16.13 ng/mL; Large White: 20.99, 16.82–25.09 ng/mL; Fig. 2).

**Fig. 3:**
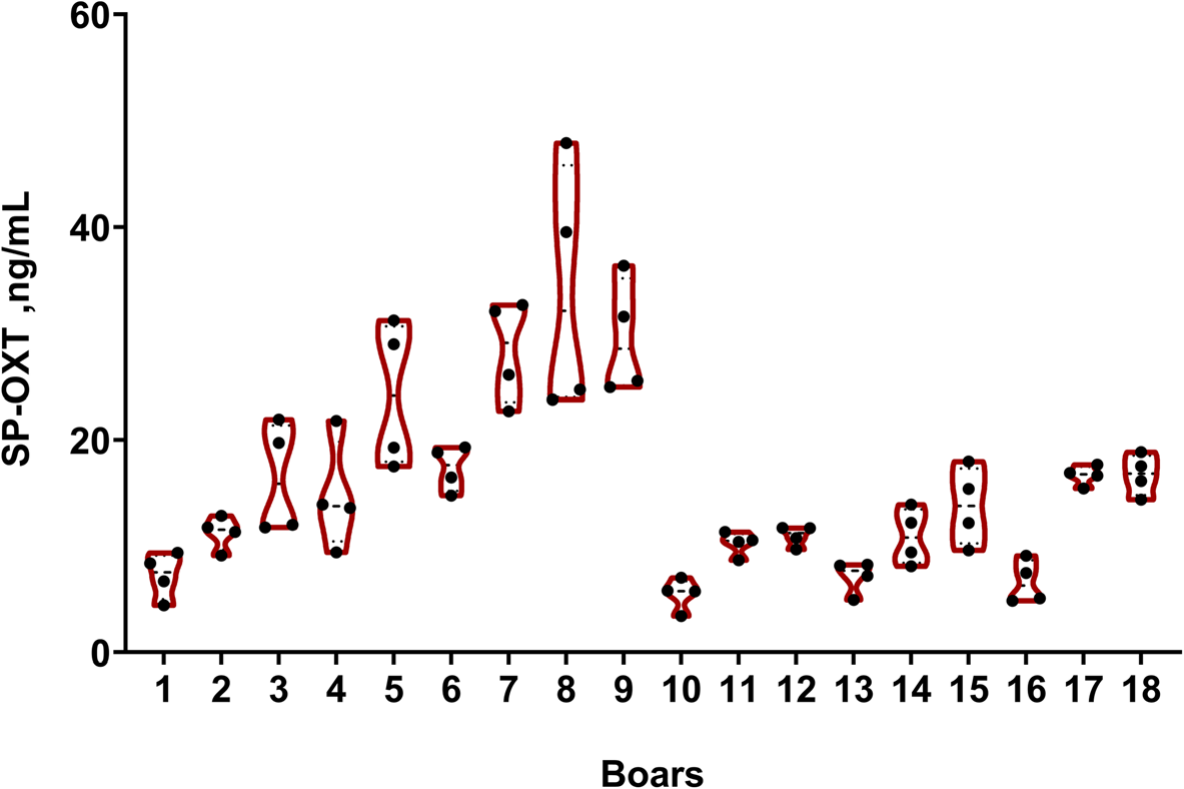
Violin plots displaying oxytocin (ng/mL) concentration levels and its distribution in seminal plasma (SP-OXT) of entire ejaculates (*n* = 72) from 18 artificial insemination boars (four ejaculates per boar). Each dot indicates individual SP-OXT values of each ejaculate, dashed lines the median and the 25^th^ and 75^th^ percentiles are indicated with dotted lines

The SP concentration of OXT varied widely (*P* < 0.001) among boars (inter-boar variability), ranging from 3.41 to 47.92 ng/mL (Fig. 3). Similarly, a wide variability (*P* < 0.001) in the SP concentrations OXT was found among ejaculates from a same boar (intra-boar variability). Despite these considerations, there was a good reliability among ejaculates within boar, as ICC (3,1) was 0.78 (0.61–0.90; 95%).

### Experiment 2: relationship between SP-OXT concentration and boar ejaculate characteristics

The Spearman correlation coefficients showed that the SP concentrations of OXT were correlated with ejaculate volume (R = 0.46; *P* < 0.01) and proportions of viable spermatozoa showing both high ROS generation (R = − 0.34; *P* < 0.05) and high plasma membrane fluidity (R = 0.35; *P* < 0.05) in the semen samples analyzed at 0 h of liquid storage (Additional File 1). Despite their significance, the Spearman correlation coefficients were below 0.5.

Semen samples were grouped (hierarchical clustering, *P* < 0.001) in two groups according to their SP-OXT, one showing the highest OXT concentrations (from 27.79 to 61.04 ng/mL, *n* = 17) and another group showing the lowest OXT concentration (from 2.91 to 24.33 ng/mL, *n* = 19). Table 1 shows the median and the 25−75^th^ percentiles of boar age, ejaculate characteristics and sperm parameter assessed in each SP-OXT group. Boar age differed (*P* < 0.05) between the two SP-OXT groups. The ejaculates with highest SP-OXT concentrations came from younger boars (median, 25−75^th^ percentiles; 16, 12.5–24.5 mos.). Similarly, ejaculates with highest SP-OXT concentrations showed higher volume (655, 598.5–693.5 mL) than those with lowest SP-OXT concentrations (608, 524–645 mL; *P* < 0.05). None of the assessed sperm parameters differed between the two SP-OXT groups, neither those assessed at 0 h nor those assessed at 72 h of storage at 17 °C.

**Table 1:** Boar age, ejaculate characteristics and sperm functional parameters in liquid-stored semen (*n* = 36) for each of the two groups of SP-samples grouped hierarchically (*P* < 0.001) according to OXT concentration

Parameter	Evaluation time-point	Median (25^th^, 75^th^ Percentiles)		***P***-value
		Low SP-OXT (2.91 to 24.33 ng/mL, *n* = 19)	High SP-OXT (27.79 to 61.04 ng/mL, *n* = 17)	
Boar age, months	–	26 (20, 33)	16 (12.5, 24.5)	**0.042**
Ejaculate volume, mL	0 h	608 (524, 645)	655 (598.5, 693.5)	**0.019**
Sperm concentration, ×10^6^ sperm/mL	0 h	161.4 (140.9, 225.3)	160.5 (139.3, 211.7)	0.679
Total sperm count, ×10^6^ sperm	0 h	99,902 (75,791, 138,077)	110,773 (82,420, 120,351)	0.825
Normal sperm morphology, %	0 h	76 (73, 90)	79 (73, 89)	0.906
Motile sperm, %	0 h	82 (75, 86)	81 (70.5, 85.5)	0.556
	72 h	76 (72, 78)	71 (64.5, 80)	0.409
Progressive motile sperm, %	0 h	52 (46, 64)	48 (39, 53.5)	0.056
	72 h	52 (40, 65)	58 (44.5, 62)	0.577
Viable sperm, %	0 h	88.4 (79.1, 90.1)	88.2 (83.8, 90.05)	0.956
	72 h	86.8 (81.7, 90.9)	86.9 (82.8, 90)	0.943
Viable sperm generating intracellular ROS, %	0 h	31.7 (23.4, 41.1)	21.5 (9.65, 42.8)	0.214
	72 h	48.3 (34.5, 62.3)	54.7 (37.95, 66.35)	0.614
Viable sperm with high plasma membrane fluidity, %	0 h	1.3 (0.8, 1.7)	1.4 (0.95, 2.2)	0.204
	72 h	1 (0.5, 5.2)	2.4 (1.05, 7.8)	0.198

### Experiment 3: relationship between SP-OXT concentration in the original ejaculate and *in vivo* fertility outcomes of extended semen samples

**Fig. 4:**
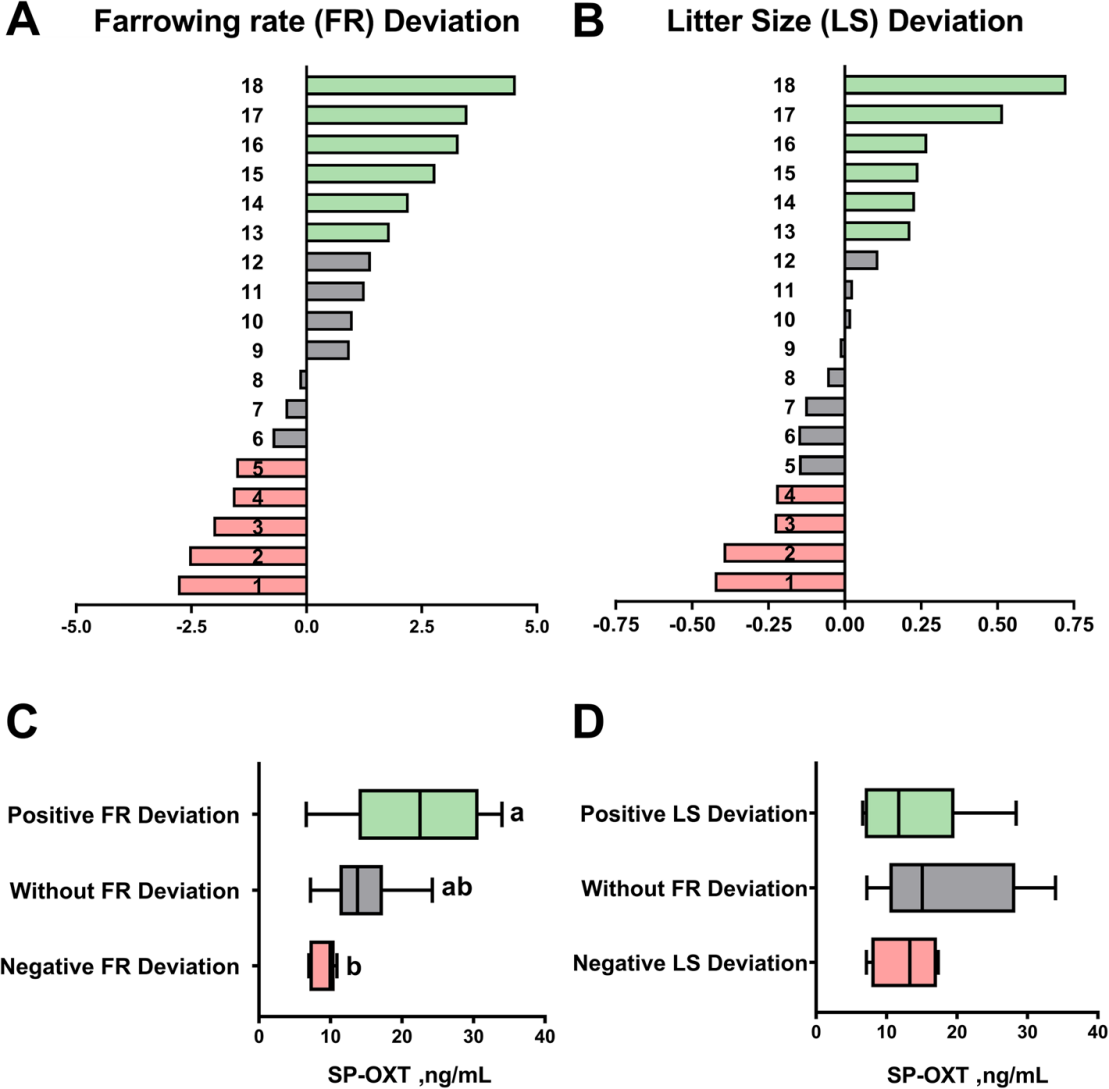
Relationship between the concentration of oxytocin in seminal plasma (SP-OXT) and *in vivo* fertility outcomes of boars used in artificial insemination programs (*n* = 18). **A-B:** Bar charts showing the deviation in farrowing rate (A) and litter size (B), measured in terms of direct boar effect, for each of the boars under evaluation. Deviations are with respect to the breed means represented by the value 0 on the X-axis. Boars were grouped (hierarchical clustering, *P* < 0.001) into three groups by having positive (green), without (grey) or negative (red) deviation. **C-D:** Box-and-whisker plot showing the SP concentrations of OXT in the ejaculates from boars showing positive, without and negative deviation in farrowing rate (FR; **C**) and litter size (LS, **D**). Boxes enclose the 25^th^ and 75^th^ percentiles; the whiskers extend to the 5^th^ and 95^th^ percentiles and the line is the median. ^a-b^ indicates significant differences (*P* < 0.05) between groups of boars in the SP concentration of OXT

The 18-AI boars included in this sub-study were those of Landrace and Large White breeds, whose semen was collected (four ejaculates per boar) over a 12-month period (one SP sample per boar every four mos.) to avoid possible seasonal effects and whose AI-semen inseminated > 100 sows. These specific 18 boars were moreover grouped (hierarchical clustering, *P* < 0.001) into three sub-groups groups according to deviations in farrowing rate: positive (from 1.81% to 7.54%, *n* = 6), without (from − 0.74% to 1.44%, *n* = 7) and negative (from − 1.53% to − 2.79%, *n* = 5) deviation (Fig. 4A). Moreover, the boars were also grouped into three further sub-groups according to deviations in total litter size: positive (from 0.22 to 0.83 piglets, *n *= 6), without (from − 0.15 to 0.11 piglets, *n* = 8) and negative (from − 0.22 to − 0.42 piglets, *n* = 4) deviation (Fig. 4B). Boars with positive farrowing rate deviation showed ejaculates with higher (*P* < 0.05) SP concentrations of OXT (mean ± SEM; 21.94 ± 4.24 ng/mL) than boars with negative farrowing rate deviation (9.04 ± 0.82 ng/mL) (Fig. 4C). Boars with positive and negative litter size deviation did not showed differences in the SP concentrations of OXT (13.69 ± 3.30 ng/mL vs 12.77 ± 2.51 ng/mL) (Fig. 4D).

## Discussion

To the very best of our knowledge, this is apparently the first study measuring the concentration of OXT in pig SP and the first report conducted in a livestock species assessing an eventual putative influence of SP-OXT concentrations on sperm reproductive performance post-AI. The data reported in the present study showed that OXT was present at measurable concentrations in the SP from all ejaculates analyzed, with the highest volume ejaculates and those from the youngest boars showing the highest concentrations. Noticeable, the results also showed that boar with best farrowing rates have ejaculates with highest SP-OXT concentrations.

The results of the first experiment revealed that OXT was present in boar SP at higher concentrations than in SP of men [26, 27] and stallions [23]. These differences were expected considering (1) variations in OXT concentrations have been reported when different OXT quantification methods are used [38], circumstance occurring among the three studies mentioned above, including the present report and (2) that differences between species in other SP components (proteins) had been previously reported and could due to species-related differences in mating strategy (vaginal vs uterine) [39]. Focusing on male pigs, the OXT concentrations measured in SP in this study were higher than those previously reported in saliva samples also collected from boars used in AI programs and measured using the same procedure [36]. This comparatively higher concentration of OXT in SP could suggest an eventual functional role in either sperm or on the female genital tract, since both in sperm (demonstrated in stallions: [40]) and in the endometrium and myometrium (demonstrated in sows: [41]) there are OXTRs. Noteworthy, the measured concentrations of OXT in single SP samples (one ejaculate per boar) varied between boars but not between breeds. These results would be in agreement with previous studies that reported variability between boars but not between breeds in other SP-proteins, such as anti-müllerian hormone or glutathione-S-transferase mu 3 [18, 42]. Moreover, variability among individuals in the SP-OXT has also been reported in humans [27]. In pigs, differences in the concentration of salivary OXT among boars has also been reported [36]. In this context, it has been reported that individual differences in OXT gene or OXTR led to a change in basal OXT concentrations in humans [43]. Therefore, it would be reasonable that the differences between boars in SP concentrations of OXT could be genetically determined. However, we do not believe that this is the case in our study. Boars of the same breed are genetically very similar, at least those used in our study. Then, the fact that there is individual but not breed differences would rule out the genetic origin. Disclose the causes of these individual variations is still challenging. Differences in libido could be a cause. López-Arjona et al. [35] showed that salivary OXT concentrations were related to libido in boars used in AI programs.

The second experiment intended to find out if SP-OXT concentrations were related to the age of boars and ejaculate characteristics and if they influenced sperm parameters from diluted semen samples stored at liquid state, such as those used in AI programs. The results demonstrated that SP-OXT concentrations were influenced by boar age, showing that the youngest boars exhibited the highest SP-OXT concentrations. These results agree with those reported by Lopez-Arjona et al. [36], who found that youngest boars had higher salivary OXT concentrations. Also, with those reported by Elabd et al. [44] in mice, who considered that OXT is a hormone that in blood plasma would be age-dependent, decreasing the concentrations as the age of individuals increased.

The SP concentrations of OXT were positively related with ejaculate volume. This relationship was expected since OXT plays a fundamental role in ejaculation, stimulating contractions of smooth muscles of the male reproductive system [20] and, thereby, facilitates spermiation, the release of fluids from the accessory glands and the passive transport of sperm [45]. The proved presence of OXTR along segments of the male reproductive tract of several mammalian species would confirm a local action of OXT [45, 46]. Specifically, in pigs has been identified OXTR in the epididymis and testis [20]. Although no studies have measured the concentration of oxytocin in the different segments of the male genital tract, it is reasonable to think that the concentration of OXT in SP was positively related to the total amount secreted by the different male reproductive tissues and, therefore, to the contractile capacity of the smooth muscle of these tissues. Then, greater contractility would result in a greater volume of the ejaculate. Mostafa et al. [27] found no relationship between seminal OXT concentration and ejaculation volume in men. The differences between men and male pigs in reproductive OXT secretion could be explained by differences in mating strategy. Specifically, vaginal deposition and low ejaculate volume in men versus uterine deposition and high ejaculate volume in male pigs.

There was no relationship between SP concentrations of OXT and ejaculate sperm concentration or total sperm count. It could be postulated that the local action of OXT would be more active in the accessory glands (adds volume to ejaculates) than in the epididymis (adds sperm to ejaculates). This hypothesis would be supported by the *in vitro* experiments carried out by Bodanszky et al. [47] in pigs, which showed that OXT is particularly active in the accessory glands, more specifically in the prostate. Our results also revealed that SP-OXT concentrations were not related to quality and sperm functionality parameters assessed in semen samples liquid stored at 17 °C during 72 h. These results agree with those of Goverde et al. [26] in men, which found that SP-OXT concentrations were not related to the motility and morphology of fresh sperm. In contrast, Mostafa et al. [27] reported negative relationships of SP-OXT concentrations and total sperm count, motility and morphology also in men. However, these relationships were supported by correlation coefficients less than 0.5, indicative of fair relationships [48]. In pigs, an empirical study reported that supplementation of diluted semen with OXT did not influence sperm motility or the integrity of plasma and acrosomal membranes in both fresh and frozen-thawed sperm [29]. Although there is evidence that mammalian sperm cells have OXTR (stallion: [40]), suggesting that SP-OXT could modulate sperm functionality, our results and the aforementioned studies would indicate otherwise, by less seminal OXT would not a major influence in sperm parameters as relevant as motility, membrane integrity or lipid peroxidation. Perhaps seminal OXT may influence other sperm functions, such as sperm capacitation. More research is required to elucidate this matter.

The last experiment focused on evaluating whether SP-OXT concentrations were related to* in vivo* fertility outcomes of liquid semen AI-doses from a selected, two-breed boars with ejaculated collected over an entire year (one per month), whose semen AI-doses were used to inseminate at least 100 sows per boar [1]. Pig fertility, being a polytocous species, is measured by the farrowing rate and also by the number of piglets born per litter (total litter size). Accordingly, the results showed that SP-OXT concentrations were positively related to farrowing rate but not to total litter size. Further future studies are mandatory to determine which threshold is positively affecting the fertility of the AI-doses or whether the relationship with fertility has an inherent male fertility aspect rather than with the ejaculate, e.g. the concentration of OXT in the ejaculate is merely an indicator of the fertility of the male, regardless of the use of AI or natural mating.

To the best of our knowledge, only a previous study, conducted in humans, evaluated the relationship of seminal OXT to male fertility and found that fertile men had lower SP-OXT concentrations than infertile men [27]. Unlike the Mostafá study [27], the boars included in our study were all fertile, to a greater or lesser extent, but fertile. In addition, the ejaculates of the Mostafá study showed clear differences in oxidative stress between fertile and infertile donors, being more accused in the latter. These differences could explain the disagreement between the results of Mostafá and ours. The positive relationship found in our study between SP-OXT and *in vivo *fertility would be supported by many empirical studies that demonstrated the positive effects of supplementing semen AI-doses with OXT on swine fertility [29–34]. The positive effect of the SP-OXT on fertility could be due to the fact that it activates endometrial as well as myometrial OXTRs, which are highly expressed during estrus and more especially during the periovulatory period [41]. This activation would lead to an increase in myometrial contractions facilitating the passive transport of spermatozoa to the utero-tubal junction thus increasing the total amount of potentially fertilizing spermatozoa in the oviductal reservoirs [28]. In this regard, Okazaki et al. [29] reported that the supplementation of pig semen AI-doses with OXT increased the number of sperm in the utero-tubal junction reservoir, having a positive impact on fertility. Moreover, SP has immunoregulatory properties, causing a favorable immune environment for sperm and embryos in the female genital tract [9]. In this matter, the role of SP-OXT could be to stimulate the secretion of prostaglandin E2 by the endometrium of sows [49], which has immunosuppressive activity and, therefore, contributes to creating a tolerogenic environment in the uterus [29]. Some of the above empirical studies found that the supplementation of AI-doses with OXT also improved litter size in addition to farrowing rates [31, 33, 34]. This increase in litter size was not found in the present study, perhaps because the SP-OXT concentrations measured in the present study were significantly lower than those used in the aforementioned studies, which ranged from 0.04 to 0.1 IU/mL. In any case, the results of these studies support our findings about the positive role of seminal OXT in porcine semen fertility.

## Conclusions

In conclusion, OXT was present in pig SP in measurable concentrations, which differed among boars and among ejaculates within boar, showing higher concentrations in young boars. The SP-OXT concentrations had not influence on functional sperm parameters in liquid-stored AI-semen. The most relevant finding of this study was that the SP concentrations of OXT were positively related to farrowing rates of a selected number of AI-boars, collected over data from 3,167 AI-bred sows. Therefore, the measurement of the SP concentrations of OXT could lead to a better selection of boars used in AI programs, contributing to the successful improvement of AI efficiency.

## Supplementary Information

**Additional file 1: Figure.** Heatmap of Spearman correlation coefficients between seminal plasma oxytocin concentrations (SP-OXT) and boar age, ejaculate characteristics and quality and sperm functionality of semen samples (*n* = 36; one semen sample per boar) stored at 17 °C during 72 h. Sperm quality and functionality was assessed at 0 h (A) and 72 h of storage (B). ** *P* < 0.01, *P < 0.05. Months: mos

## Data Availability

The datasets from the current study are available from the corresponding author on reasonable request.

## Abbreviations

AI: Artificial insemination
CASA: Computer assisted sperm analyzer
CM-H_2_DCFDA: 5- (and 6-) chloromethyl-20,70-dichlorodihydrofluorescein diacetate acetyl ester
DCF: 2′,7′-Di-chlorofluorescein
DMSO: Dimethyl sulfoxide
H-42: Hoechst 33342
ICC: Intra-class correlation
M-540: Merocyanine 540
Mos: Months
PI: Propidium iodide
PNA-FITC: Fluorescein-conjugated peanut agglutinin
OXT: Oxytocin
OXTR: Oxytocin receptor
PBS: Phosphate buffered saline
ROS: Reactive oxygen species
RT: Room temperature
SEM: Standard error of the mean
SP: Seminal plasma
