# Supplementary figures and images for: Oxytocin in pig seminal plasma is positively related with in vivo fertility of inseminated sows
Source: J Anim Sci Biotechnol. 2021 Sep 13;12:101. doi: 10.1186/s40104-021-00620-z (PMC8436503; doi:10.1186/s40104-021-00620-z)

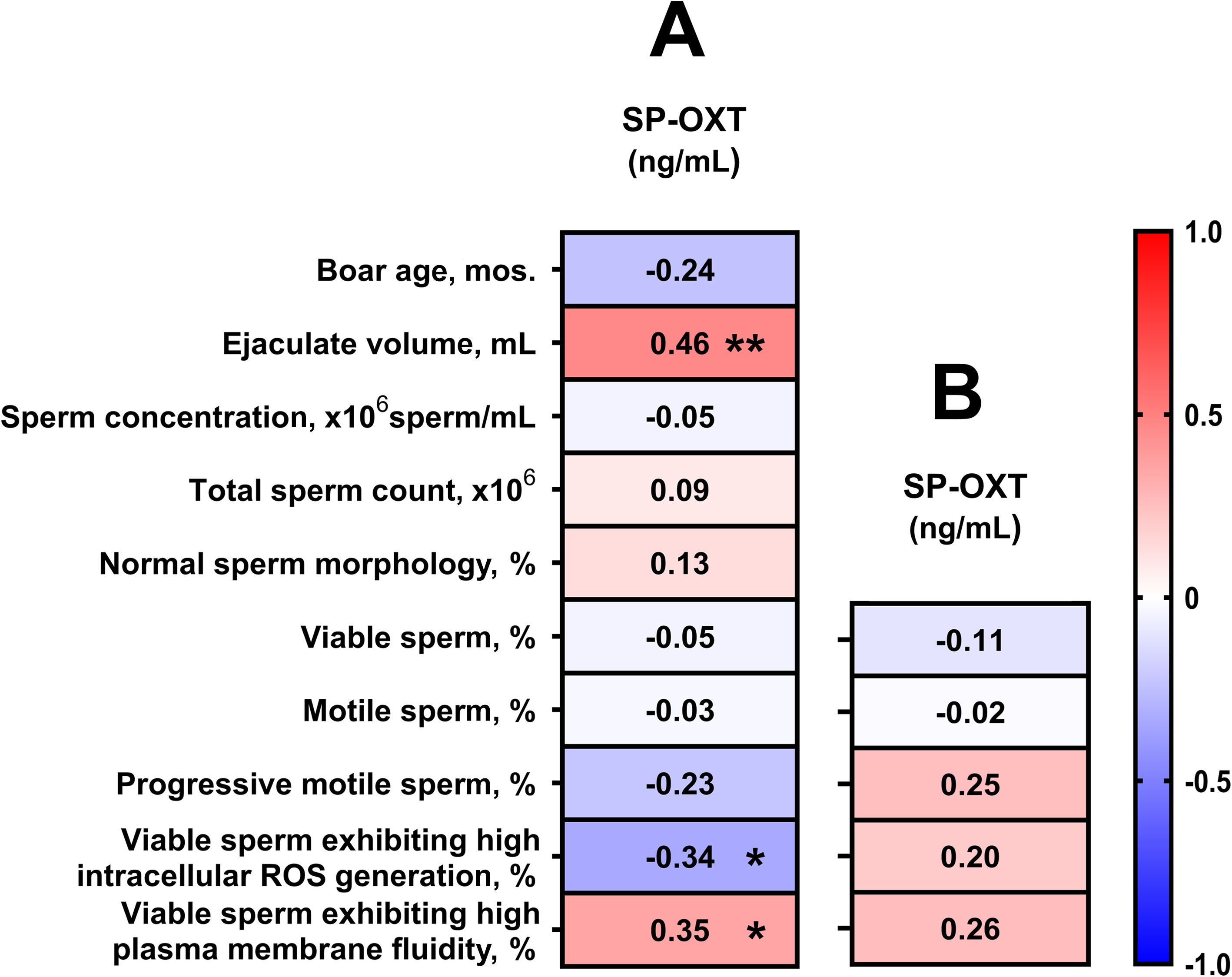

Supplement: Supplementary file 1 — Additional file 1: Figure. Heatmap of Spearman correlation coefficients between seminal plasma oxytocin concentrations (SP-OXT) and boar age, ejaculate characteristics and quality and sperm functionality of semen samples (n = 36; one semen sample per boar) stored at 17 °C during 72 h. Sperm quality and functionality was assessed at 0 h (A) and 72 h of storage (B). ** P < 0.01, *P < 0.05. Months: mos [file 40104_2021_620_MOESM1_ESM.tif]
